# Overcoming therapeutic nihilism. Breaking bad news of amyotrophic lateral sclerosis—a patient-centred perspective in rare diseases

**DOI:** 10.1007/s10072-022-05931-1

**Published:** 2022-02-12

**Authors:** Stanisław Maksymowicz, Maria Libura, Paulina Malarkiewicz

**Affiliations:** 1 Department of Psychology and Sociology of Health and Public Health, School of Public Health, Collegium Medicum of the University of Warmia and Mazury, Olsztyn, Poland; 2 Instytut Terapii Komórkowych S.A., Olsztyn, Poland; 3 Medical Education and Simulation Department, School of Medicine, Collegium Medicum of the University of Warmia and Mazury, Olsztyn, Poland; 4 Department of Obstetrics and Gynaecology, School of Medicine, Collegium Medicum of the University of Warmia and Mazury, Olsztyn, Poland

**Keywords:** Breaking bad news, Amyotrophic lateral sclerosis, Diagnosis, Communication

## Abstract

Amyotrophic lateral sclerosis (ALS) is a rare, incurable, and fatal neurodegenerative disease with median survival time from onset to death ranging from 20 to 48 months. Breaking bad news about ALS diagnosis is a challenging task for physicians and a life-changing experience for patients. Several protocols for delivering difficult information are available, including SPIKES and EMPATHY. Our goal was to assess to what extent these guidelines are followed in Polish ALS patients’ experience as well as to identify any other patients’ preferences not addressed by the guidelines. Participants of our study were recruited via a neurology clinic. Twenty-four patients with confirmed ALS diagnosis were interviewed using in-depth interview and a self-constructed questionnaire: 9 females, 15 males in age ranging from 30–39 to 60–69. The analysis showed a pattern of shortcomings and fundamental violations of available protocols reported by ALS patients. Patients also had to deal with therapeutic nihilism, as they were perceived as “hopeless cases”; unlike in oncological setting, their end-of-life needs were not accommodated by some standard schemes. As a conclusion, we recommend using extended breaking bad news protocols with special emphasis on preparing a treatment plan, giving the patient hope and sense of purpose, offering psychological support and counselling directed to patients and caregivers, and providing the patient with meaningful information about the disease, social support, treatment options, and referral to appropriate health care centres.

**Supplementary Information:**

The online version contains supplementary material available at 10.1007/s10072-022-05931-1.

## Introduction

Amyotrophic lateral sclerosis (ALS), known also as Charcot or Lou Gehrig disease, is a fatal neurodegenerative disease that affects both central and peripheral neurons, leading to progressive paralysis. ALS is a rare disease [1]. In most cases (90%), it manifests itself sporadically without any known cause [2].

Therefore, from the patient’s point of view, the diagnosis is usually unexpected and comes as a shock. The way in which a message of an incurable disease is delivered has high impact on both the patient and physician [3]. It will also shape the attitude of the patient toward the disease and toward symptomatic treatment measures available [4]. While most breaking bad news protocols have their roots in oncological context, e.g. SPIKES [5], they seem well suited to other life-changing diagnoses as well [6]. As in oncological context, patients with rare diseases also value ensuring that a timely follow-up is planned; offering informational sheets about the diagnosis; offering contact information of support organizations, with some patients preferring patient support groups while others preferring counsellors; and conveying a sense of determination to aid the patient through the diagnosis [ibid.].

The purpose of the present study was to examine the way in which patients in Poland receive the information about the diagnosis of ALS to propose patient-centred recommendations that consider patients’ perspective. Our goals were twofold: first to assess the extent to which diagnosis deliver practice conforms to established protocols, second to identify any possible gaps in patients’ needs and suggest supplementary recommendations for rare diseases.

## Materials and methods

The study, approved by The Scientific Research Ethics Committee of the University of Warmia and Mazury in Olsztyn (decision no. 5/2018), was conducted using the PAPI (Paper and Pen Personal Interview) method between February and June 2018. The subjects were private clinic patients (Instytut Terapii Komórkowych – Cell Therapies Institute in Olsztyn, Poland) with diagnosed ALS. The ability to communicate—even if with the support of a caregiver—and good mental health constituted inclusion criteria. Thirty patients were qualified for the study, but ultimately, information was collected from 26 subjects. Two surveys were rejected due to the incompleteness. Twenty-four fully completed interviews were subjected to final analysis, which is a considerable result, given the fact that ALS is a rare disease (Table 1). The interview questionnaire consisted of 30 closed questions (including demographic questions, questions about the diagnostic path, and opinions on the diagnosis), as well as 5 open questions (related to the patient’s experience in each area). The questionnaire translated from Polish original is included in Supplement 1.

**Table 1:** Demographic data (*N* = 24)

	Gender		
Age	Female	Male	Sum
30–39	0	1	1 (4.16%)
40–49	3	5	8 (33.3%)
50–59	3	6	9 (37.5%)
60–69	3	3	6 (25%)
**Sum**	**9**	**15**	**24 (100%)**

As a result, a demographically non-homogeneous sample was obtained. In terms of age, the group was divided into 3 cohorts: 40–49 (33.3%), 50–59 (37.5%), and 60–69 years (25%). In addition, there was one patient under 40 years of age in the sample—exceptionally young for this disease. The distribution of the “gender” variable was 9 (females) to 15 (males), which corresponds to the distribution occurring in ALS disease, characterized by the predominance of men, with 2:1 male to female gender ratio [1]. The distribution of the variable “place of residence” in the sample was also relatively large, with a small advantage of the big city (over 250 thousand).

## Results

The process of communicating the diagnosis to patients with ALS is both a challenge for the physician and an extremely difficult experience for the patient [7, 8]. In our study, we focused on the experience of the latter. Based on the interviews, we were able to identify some fundamental problems and challenges related to this process.

### Diagnosis setting

**Fig. 1:**
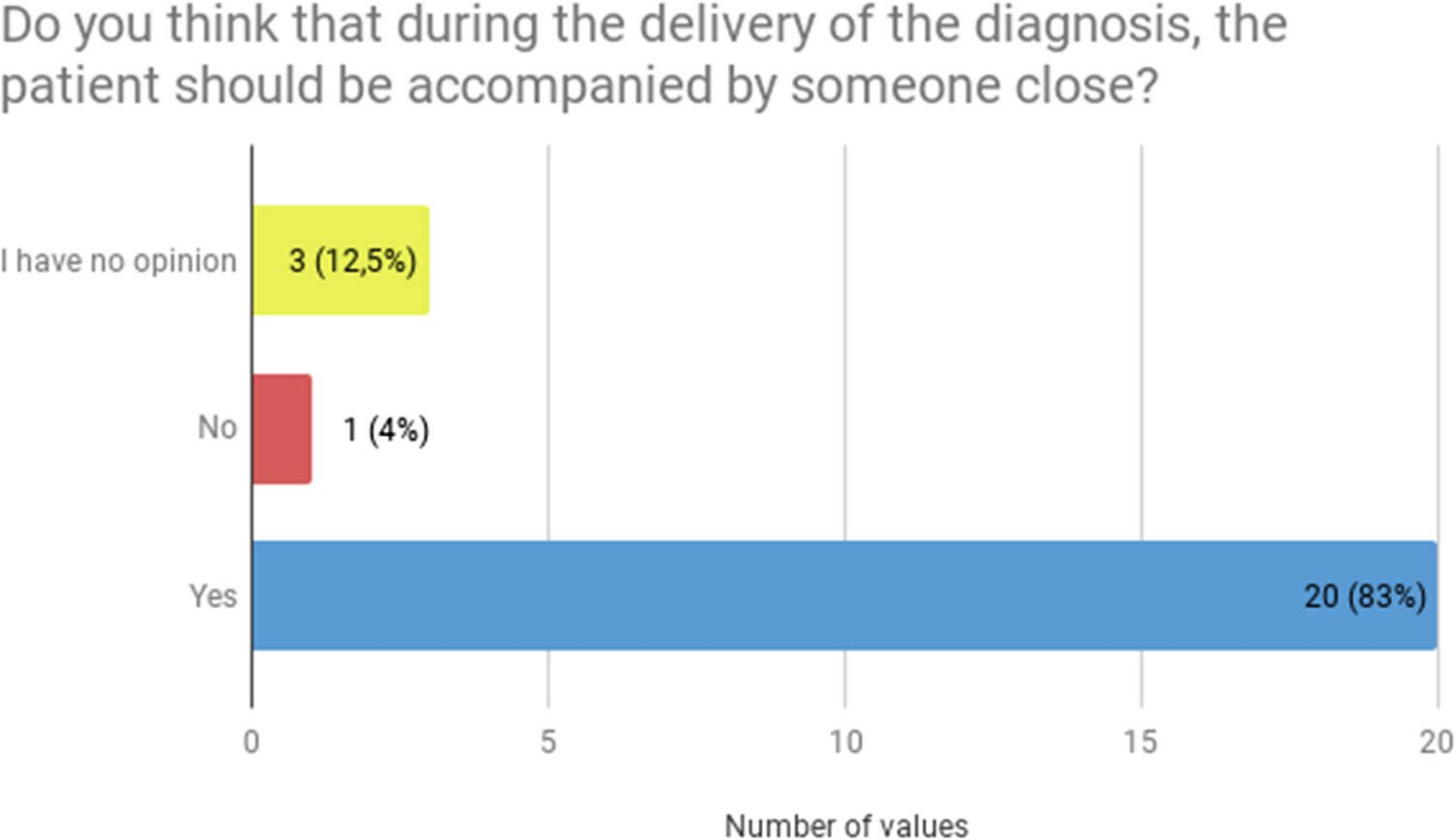
Patients’ preferences concerning having someone close during diagnosis delivery (*N* = 24)

**Fig. 2:**
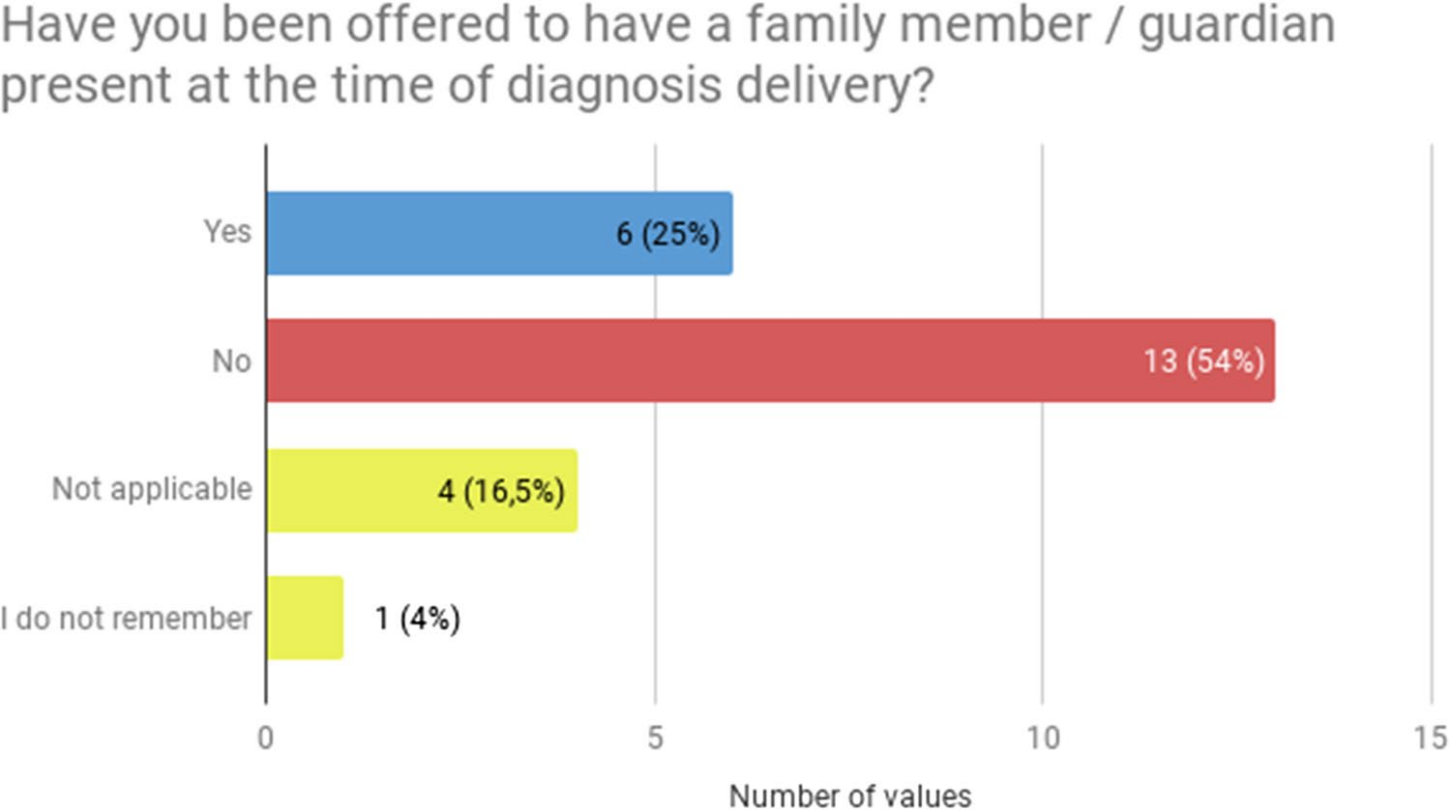
The actual practice of offering the patient to have someone close during diagnosis delivery (*N* = 24)

First, a common issue reported by the respondents was lack of someone close to the patient during diagnosis delivery. Majority of patients stressed that during the delivery of the diagnosis, the patient should be accompanied by someone close (20, 83.3%, Fig. 1). But only a quarter (6, 25%) have been offered to have a family member/caregiver present at the time of diagnosis delivery (Fig. 2).

Second, patients were concerned with the setting of diagnosis delivery. Most often it was disclosed in the doctor’s office, but often also in a hospital room with other patients present (in 7 cases, 29%) or even in the corridor, random utility room, or at the door of physician’s office. One patient found out about his disease by telephone, while receiving his EMG (electromyography) results. The setting where diagnosis delivery takes place is not trivial and affects the patients’ perception and well-being heavily.

Here is how the situation was described by one person who was informed about the diagnosis in a random hospital room without any proper preparation: “You are dying, there is no cure, there is no point in doing anything else—doctor said. I was crying in the corner; a stranger was consoling me. The description of illness was given in a cold, brutal way”. Another person mentioned that he was given the diagnosis at the admission room in a hospital. His wife was asked to leave. All he heard was that there is nothing more to be done. And the patient is going to “die by suffocation”. The patient was then left alone in the room and in a few minutes asked to leave it, because the room was needed for next person.

### Lack of empathy

Listening to and understanding the patient’s emotions, crucial skill in conveying difficult information, was usually not exhibited by the doctor at all. This is another problem identified in our study. This is what one patient recalls: “I cried, it was a moment, but I couldn’t recover. The doctor did not describe the course of the disease, but waited for us to make the diagnosis ourselves: ‘The conductivity showed… you can guess what it is …’ I cried and left, unable to talk. Later there was no chance for a conversation”.

Only in rare cases did the doctor try to convey the diagnosis in the most gentle and hopeful manner. One patient recalls this moment: “You have a lot of time, maybe medicine will come up with something by then”. Another patient describes the situation with a young doctor (resident), who was very afraid of giving the diagnosis. She prepared a printed sheet to give to the sick person with information about disease duration: life from 2 to 3 years, sometimes longer to 10. “There is no medicine, you must enjoy life now, grab it with your handfuls”—the patient heard from the doctor.

Patients asked if doctor said something that was particularly useful, compassionate, or encouraging during the delivery of the diagnosis, mostly denied it (18, 75%). Only in one case, such a harsh treatment of the diagnosis turned out to be useful for the caregiver of the patient: “It was an excitement to have to do something about it now, it was an impulse, a feeling to act”.

In 6 cases, patients indicated specific elements of the conversation that were emotionally or informatively helpful for them. These included the following:

- a sincere conversation related to religion (the patient was a priest),
- a doctor who “wanted the diagnosis to be different” and conducted all tests that the family wanted additionally (also, the psychologist was invited to consult the patient right after the interview),
- the head of the hospital ward who looked for different variants and tried to be gentle when he gave the diagnosis, compassion, and long and gentle conversation.

On the other hand, patients were asked if the doctor said anything unnecessary, improper, or soulless during the delivery of the diagnosis, also mostly denied (17, 70%). Among 7 patients (29%) who have experienced such behaviour, there were statements pointing to several problems:

1. Therapeutic nihilism:
2. “A warning that she (wife – the patient – ed.) will have to go to a hospice”.
3. “We feel sorry for the family, a lot of suffering ahead of you”.
4. “The doctor suggested psychotropic drugs for me and my family (after the diagnosis)—so that they would positively accept the diagnosis”.
5. “ [Clinic name] (where diagnosis was made—ed.) deprived me of opportunities and hope, it was quickly said what and how and goodbye. It was also there that the word ALS was mentioned for the first time. And the statement: 2–5 years, death by suffocation”.
6. Lack of control over body language:
7. “The doctor—the head of the clinic—the day after the EMG has been performed, comes with results and says with a smile on her lips that it is ALS. Nothing more. I remember that smile when she said, it was ALS”.
8. Taking advantage of the situation:
9. “The doctor said, that on a private visit, he will tell me how to live with it (so we didn’t go). He did not want to talk in the hospital—so the conversation in the corridor took place (about diagnosis)”.
10. No reference to patient’s emotions and prior knowledge:
11. “The doctor (…) wanted to discharge me as soon as possible. (…) The conversation about the disease was during examination. The doctor only said that I had read about this disease before. I had a lot of notes and questions—I got the answer, but the doctor fidgeted, didn’t take it well, reacted negatively to the next questions. This is a shortcut approach and relying on the knowledge I have acquired on my own”.
12. “Very casually [the doctor conveyed the information] in 2–3 sentences and sent him home, even the assistant came and asked if she could help drive him (patient – ed.) home. But there was no conversation”.

### Information gap

**Fig. 3:**
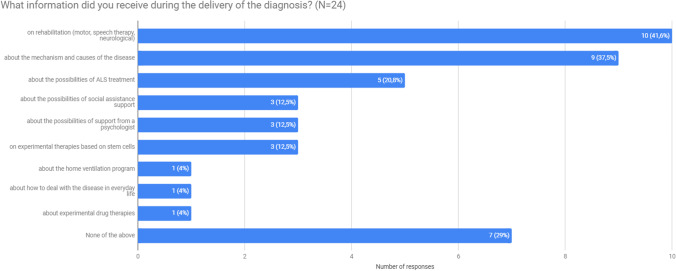
Information received at the time of diagnosis (*N* = 24)

Another often recurring theme was lack of information about the disease, its progress, and further action plan. For 75% (18) of patients, the diagnosis was explained in an understandable way. However, most patients did not receive any additional materials about the disease and treatment from their doctor (75%, 18). Three patients (12.5%) received a contact to a doctor dealing with ALS (including experimental treatment) and another 3 (12.5%)—some printed materials about the disease. Only 3 patients found specific materials as particularly useful: contact to a doctor (2 patients) and printed materials about the disease (1 patient). One patient received an Internet address of an ALS association. When asked what information they received, 29% (7) of respondents said that they didn’t receive any. List of information types provided during the diagnosis is shown in the Fig. 3.

Further, the assessment of the usefulness of the information obtained was not high. When asked if, in retrospect, patients missed some information that would have been helpful, most responded positively (58.3%, 14). Several basic problems can be identified from the statements of patients:

1. Self-diagnosis: some patients knew a lot about the disease from the Internet, they later had their self-diagnosis confirmed in the hospital—in those cases consultation at the time of diagnosis delivery often lacked added value.
2. Too general information about the disease: patients had to do a lot of research on their own related to practicalities of disease management as well as disease progression and symptoms.
3. Lack of information about care and support options: patients did not know where to turn for help and what to do next; many complained about the flow of information in health care system regarding ALS.
4. Lack of information about possible options at an early stage, such as clinical trials or for example experimental treatment.
5. Lack of plan: patients complained about not knowing what to do next with the disease, how to follow-up with rehabilitation, how to live with the disease, how to find specialised centres, receive consultation when needed, obtain home ventilation, and what kind of diet to follow. Such information comes mainly from other patients.

One patient said that he didn’t even want to hear this information. He decided that if medicine has no cure for this disease, patients should not be informed about it. Thanks to this, they could safely die without waiting for the next stages of the disease (paraphrase based on the words of the patient). This is an exceptional situation, but it coincides with the indications of the SPIKES protocol [5], which emphasizes that one should not force information that the patient does not want to hear or for which he is not ready.

Here, we touch on another important issue: when the diagnosis should be communicated to patients. Most of the patients stressed that they would like to know their diagnosis whenever there is a suspicion of disease (45.8%, 11), another 25% (6) found that they would rather know after getting a complete diagnosis. However, getting to know the disease as quickly as possible was important for most patients.

### Disease prognosis

As for the delivery of disease prognosis, an analysis of patients’ responses revealed again a wide range of mixed experiences. Some patients received a comprehensive description of what may await them, as well as what the course of the disease may look like. As expected, the time allocated to discuss and ask questions regarding different therapeutic options was greatly appreciated by the patients. In concord with answers to the question on information scope, patients expected guidance in terms of next steps to be taken following the diagnosis. These, however, were in many cases limited to a referral to a hospital or a hospital outpatient clinic. In retrospect, patients were critical of the narrow scope of the advice they were given; as one patient put it: “the doctor neither referred us to a speech therapist, nor advised about the need for physiotherapy or other issues, he did not help us organise care—we had to figure everything out on our own”. In the words of another one, “I would not attach so much importance to the diagnosis, as to further management”.

A recurring theme brought up by patients dissatisfied with information on prognosis was the doctor’s focus on the neurobiological aspects of the disease rather than on the practicalities of living with the disease. Some doctors “skipped” the period of living with the disease by briefly indicating its expected length and focused on the manner of death: “in the worst-case scenario you have 3 to 6 months” or “you have 2–5 years before death by suffocation”. Others preferred more general statements over such precision, yet to a similar effect: “the course is rather short” and “the perspective is rather bleak”. In one patient’s memory, the clinical encounter boiled down to the following message: “You will die by suffocation”.

Some patients shared vivid memories of the doctor’s fears surrounding the diagnosis delivery. “It was tragic”, commented a patient who felt the doctor was afraid to break the difficult news. Others observed that the doctor was sorry and seemed to feel awkward. In some cases, the patients felt the doctors avoided them physically, not to be confronted with the task of a difficult encounter. Others sent the patient away, saying they knew nothing about the disease. A commonplace that “what could be done has already been done” was often quoted by the patients yet another avoidance strategy. When the diagnosis was delivered by young doctors, the patients reported the uneasiness of the physician, at the same time appreciating the efforts and pre-preparation to the meeting.

Variability of clinical course of ALS was presented by some physicians as the source of hope for the patients. A few doctors used it to leverage the information on the incurable character of ALS and infuse some hope into the diagnosis. In other instances, though, it seems to have been employed as a way of avoiding discussion of the disease course. In one case, for example, the patient and his family admitted they had no idea what the disease was like, yet they were only told it was incurable and saved any further details except for the information that the course of the disease was hard to determine.

As a growing number of patients is using the Internet to search for medical information, it is worth noting that some doctors warned patients not to do it. This was sometimes done in good faith, to help patients avoid simplified and often unfavourable descriptions of the disease. Yet, confronted with the scarcity of information, the patients felt the Internet was the main source of disease-related knowledge. At the same time, they admitted they would prefer the information to be delivered by the physicians themselves.

Patients found consolation in examples of well-known public figures suffering from ALS, especially in cases of slow-progressing disease that did not stop the affected person from leading an active life, such as Professor Stephen Hawking. Some valued information on experimental treatment options, as it gave them a cause for action. Many stressed that they wanted to know the truth (“it is not right to lie”), but also not to be left without any hope, even if that hope meant prolonging life in a relatively good state.

## Discussion

The main goal of our study was to explore patients’ experience with ALS diagnosis delivery. Patients’ expectations seem to match the established guidelines for breaking bad news, which are incorporated into clinical skills curricula. Such tools commonly include protocols used primarily in oncology like SPIKES [5] or EMPATHY [9]. Overall, they seem well suited to other life-changing diagnoses as well [6]. However, both the nature of rare diseases, especially incurable and fatal, and the existing support structures, require certain modifications.

Patients generally believe that the most experienced physician should inform them about the diagnosis and details of their medical condition [10]. This seems particularly true in rare diseases, where even specialists in a given field may have a very limited understanding of the course of disease and treatment options.

As reported in some previous studies, patients with rare diseases require more effort to assure a timely follow-up; offering information sheets about the diagnosis may be needed, given the scarcity of available resources [6], for non-English speakers in particular. Offering contact information of support organizations is key, as they tend to have most up-to-date information on support available in rare conditions, while conveying a sense of determination to aid the patient through the diagnosis is necessary in order to maintain reasonable hope [ibid.]. Patients expect professionals to demonstrate empathy and compassion, provide a balanced description of conditions, and make referrals for further care and support [11]. This can minimise the negative psychological impact of the news and maximise psychological well-being. Crowe et al. conducted a study on information and communication needs in rare diseases in Northern Ireland. Their findings show that improving medical care in rare diseases requires coordinated efforts of researchers, practitioners, and policy makers aimed at closer cooperation with patients, carers, and rare disease advocates. The key problem with rare diseases was reaffirmed: difficulties with finding the right health and social care information [12]. Patients’ personal experiences point to the pivotal role of individual health care professional’s engagement, as showed by Jeppesen et al., in their work on ALS. Yet, the perspectives of patient and professionals on information about disease and prognosis diverged significantly [13].

These findings are in concord with the 2017 study conducted by the EURORDIS—Rare Disease Europe Organization. Based on an international survey, it demonstrated the severity of impact of rare disease on everyday life and scarce guidance provided by health care professionals. It is not only low frequency of occurrence but also the complexity of such diseases and their variable and often evolving presentation that pose substantial challenges. Rare diseases demand a lot of dedication from both the patients themselves and their carers, often requiring substantial changes in everyday and professional lives in order to accommodate for complex needs. Unfortunately, because of gaps in support structures, both in psychological and medical care, many people with rare diseases face the problem of social isolation. This leads to a reduction in the quality of life, especially in the area of social and psychological functioning [14]. Relevant information provided in an appropriate manner at the time of diagnosis is thus key to both medical care and social support a patient will seek.

ALS is a rare disease that inevitably leads to the death of the patient. Giving such a diagnosis is stressful and challenging for the doctor, which may lead to errors in the diagnosis delivery, causing irreversible emotional consequences for the patient. The way in which ALS diagnosis is communicated to the patients is a key factor determining their initial reaction [15]. Communication skills are thus crucial, as is experience with treating patients with this particular disease: providing information to the patient requires preparation, time, intimacy, tact, and respect. Unfortunately, systematic research related to effective communication regarding end-of-life for persons with amyotrophic lateral sclerosis are still lacking [16]. However, it is possible to highlight some important elements.

Rabow and McPhee proposed the mnemonic of ABCDE consisting of advanced preparation, building a therapeutic environment, communicating well, dealing with patient and family reactions, as well as encouraging and validating emotions [17]. Similar steps are envisaged in SPIKES and EMPATHY protocols. There is no single ideal method for communicating the diagnosis to the patient, but guidelines provide a good guidance to the task. The experiences of our patients confirm that the conversation about the diagnosis should be carried out by experienced doctors, especially with patients with such condition as ALS [18]. When the doctor was not properly experienced, patients felt the lack of knowledge and preparation of physicians which significantly worsened their mental state. Therefore, as the accounts of the patients’ show, information about diagnosis should be communicated in a clear and empathetic manner, including prognosis and all therapeutic possibilities. It is unacceptable to leave patients without full information and an indication of further treatment course. Also giving reasonable hope seems to be paramount [19]. An important supplement to the information provided in the diagnosis are those obtained from the outside. Especially patients’ organizations are very helpful. Sometimes such organizations are the best source of meaningful information on disease and care options. Other patients provide inspiration, compassion, and hope [20].

Interestingly, the doctors themselves admit their limited ability to convey bad news. Often, the knowledge gained during studies is insufficient, and the knowledge gained during work is sometimes only based on the experience acquired through observations [21].

For this reason, the problem of communicating difficult diagnosis has strong implications for medical school curricula. In Poland, at medical universities, this subject is most often offered in the form of optional courses. An interesting example of the compulsory course for medical students is the University of Leipzig, where a curriculum for teaching communication skills between patient and physician has been established, based on the COMSKIL CST program. The German “Masterplan Medizinstudium 2020” adopted in 2017 by the German Federal Ministry of Health and the Federal Ministry of Education and Research (BMG) assumes the need to educate medical students to communicate effectively with patients in various clinical situations [22]. The importance of incessant education to communicate was shown in a study conducted at the Faculty of Medicine of the University of Porto, Portugal, which was aimed to determine the level of communication skills after a 4-month communication skills course for students. Skills were evaluated after 3 years. A slight deterioration in the way of communicating with the patient was noticed, which proved the necessity to constantly update these skills [23]. The British NHS (National Health Service) can be considered as a model in which good communication with the patient is recognized as an important goal that can lower treatment costs and improve treatment outcomes. On June 2020, NHS has published report “Improving communication between health care professionals and patients in the NHS in England. Findings of a systematic evidence review and recommendations for an action plan”. The report broadly discusses the need for good communication with the patient. Assuming an annual training system for medical personnel, it also emphasizes the economic aspect of this venture [24].

The attested shortcomings in diagnosis delivery in ALS seem to result from limited knowledge about a rare disease even among specialists—neurologists in this case. As we noted during interviews with our respondents, since specialists are likely to encounter ALS patients only a few times throughout their careers, the first case usually comes quite as a surprise. But the learning process cannot afford accidental actions, in diagnosis delivery.

The importance of the therapeutic relationship with the health care professionals and the value of the caring act come to the fore in positive accounts. Patients valued being assured of continuous support, no matter what the course of disease would be. The ongoing interest contributed to the patient’s well-being, as expressed by invitations to periodic health assessment. Patients appreciated if the information about prognosis was passed on gently, with probing questions assessing how much a given person can handle. In one of our studied patients’ testimonies, “such psychological help is even more important than the actual treatment”.

Our observations coincide with those of other studies [25]. They indicate among others the uniqueness of the situation of providing a diagnosis of ALS and other rare diseases, the need to pay attention to the patients’ emotions, use a psychologist for this process, provide information that is true, but not devoid of hope, listen to the patients’ reactions, and preparing a proposal for a disease management plan. All elements are necessary for this process to minimize the negative impact on the patient’s health.

## Conclusion

Based on the results of our study and a review of other studies, we propose a list of patient-centred recommendations which should be used in the process of delivering difficult diagnosis in rare diseases:

1. Protocols for breaking bad news, such as SPIKES protocol, should be used while delivering the diagnosis [5, 26].
2. In particular, the right approach includes careful attention to the patient’s emotions, appropriate setting of the conversation, and providing the patient with the opportunity to conduct a conversation in the company of someone close.
3. The physician should also be equipped with a treatment plan with information about the rehabilitation and physiotherapy and be prepared to explain health and care challenges. Even if it is only a palliative treatment that is left, it should be presented so that the patient feels taken care of, which allows to maintain hope and sense of purpose.
4. Psychological support and counselling directed to patients, to caregivers, and to physicians should be provided in every step of the disease, starting from the diagnosis delivery [27] to using clinical tools as ALSFRS-R (an instrument for evaluating the functional status of patients with ALS) which should be conducted face-to-face with patients, rather than completed by the patient alone. Such an action can improve communication with patients and reveals the needs with changes in health conditions [28]. At this point, maintaining regular contact with patients and their caregivers is also of the essence.
5. Providing the patient with useful information about the disease, social support opportunities, possible treatment options, rehabilitation [29], clinical trials, or approved experimental therapies [30–32] is key, as is referral of the patient to appropriate health care centres.

## Supplementary Information

Below is the link to the electronic supplementary material.

Supplementary file1 (PDF 146 kb)

## Data Availability

The datasets are available from the corresponding author on reasonable request.
