# Supplementary material for: Overcoming therapeutic nihilism. Breaking bad news of amyotrophic lateral sclerosis—a patient-centred perspective in rare diseases
Source: Neurol Sci. 2022 Feb 12;43(7):4257–65. doi: 10.1007/s10072-022-05931-1 (PMC9213364; doi:10.1007/s10072-022-05931-1)
Supplement: Supplementary file 1 — Supplementary file1 (PDF 146 kb) [file 10072_2022_5931_MOESM1_ESM.pdf]

Supplement 1. Translated questionnaire.

PATIENT ID: …………….. DATE: ……………………………… INVESTIGATOR ID:

TEST WITH: ☐the patient him/herself ☐patient with some assistance of the carer ☐the carer with the patient 's participation ☐ the guardian only

METHOD: ☐in person ☐by phone ☐via the Internet ☐traditional correspondence

M. DEMOGRAPHIC QUESTIONS

M.1. Age<br>○ 18-29<br>○ 30-39<br>○ 40-49<br>○ 50-59<br>○ 60-69<br>○ 70 years or more	M.3. Location<br>○ Rural area<br>○ city up to 50,000 residents<br>○ city up to 100,000 residents<br>○ city up to 250,000 residents<br>○ a city with over 250,000 residents
M.2. Sex<br>○ woman<br>○ man	M.4. Education<br>○ elementary<br>○ junior high school<br>○ high shool<br>○ higher

A. DIAGNOSTIC PATH

A.1.1. When did the first symptoms occur?<br><br>A.1.2. Description of symptoms:	
A.2.1. When was first diagnosis established (any diagnosis, not necessarily the correct one)?<br><br>A.2.2. What was the diagnosis?<br><br>A.2.3. Where? By whom? (physicians specialty)	
A.3.1. When was subsequent diagnosis established?<br><br>A.3.2. What was the diagnosis?<br><br>A.3.3. Where? By whom?	
A.4.1. When was subsequent diagnosis established? (if applicable):<br><br>A.4.2. What was the diagnosis?<br><br>A.4.3. Where? By whom?	
A.5.1. When was the final diagnosis established(SLA):<br><br>A.5.2. Where? By whom?<br><br>A.5.3. Self-diagnosis:	YES/ NO

(time from first symptoms to final diagnosis: …………….. number of incorrect diagnoses ……………..)

B. DIAGNOSIS DELIVERY

B.1. Was it suggested that a family member / guardian could participate in the interview?
- o Yes
- o No
- o I don't remember o does not apply (there was such a person)

B.1.1. Do you think it is good to have someone close present during the diagnosis delivery?
- o Yes
- o No
- o I have no opinion

B.2. The conversation took place:
- o In the doctor's office
- o In the hospital room
- o In the hospital corridor / clinic
- o Other, what: …

B.3. Was the diagnosis communicated to you in an understandable way?
- o Yes
- o No
- o Hard to say

B.4. How was the course of the disease / the prospect of living with the disease described to you?

……………………………………………………. ……………………………………………………. …………………

… …………………………….. ……………………………………………………. …………………………………

……… ………… ……………………………………………………. ……………………………………………

…. ……… ………………………………………… ……………………………………………………. ……………

……………… …………………….. ……………………………………………………. ……………………………

…………………… …. ……………………………………………………. ……………………………………………

B.5. Which of the following tests were performed prior to diagnosis:
- o EMG
- o Magnetic resonance imaging
- o Genetic
- o Neurological
- o Other what

B.6. During the delivery of the diagnosis, did you hear anything that was particularly helpful, uplifting or reassuring?
- o Yes Go to question B.6.1.
- o No Go to question B.7.
- o I don't remember Go to question B.7.

B.6.1. What words and / or behaviors of the doctor (other members of the healthcare team) were particularly supportive, helpful, uplifting or reassuring for you?

……………………………………………………. ……………………………………………………. …………………

… …………………………….. ……………………………………………………. …………………………………

……… ………… ……………………………………………………. ……………………………………………

…. ……… ………………………………………… ……………………………………………………. ……………

……………… …………………….. ……………………………………………………. ……………………………

…………………… …. ……………………………………………………. ……………………………………………

B.7. During the diagnosis, did you hear anything that was unnecessary, inappropriate or soulless?
- o Yes Go to question B.7.1.
- o No Go to question B.8.
- o I don't remember Go to question B.8.

B.7.1. What words and / or behaviors of the doctor (other members of the medical staff) were, in your opinion, unnecessary, inappropriate, soulless?

……………………………………………………. ……………………………………………………. …………………

… …………………………….. ……………………………………………………. …………………………………

……… ………… ……………………………………………………. ……………………………………………

…. ……… ……………………………………………. ……………………………………………………. ……………

B.8 . During the interview during which you were given the diagnosis, did you receive (if any of the following items are indicated, go to B.9., If not go to B.10):

- leaflets / other printed material about the disease
- link to the website of the patients' association / foundation / support group
- contact to patient' association / foundation / support group
- contacting a doctor specializing in ALS treatment (including clinical trials etc.)
- I have not received any material about the disease
- I do not remember
- Other: ….

B.9. Which materials and sources of information turned out to be particularly helpful in the first months after diagnosis?

- leaflets / other printed material about the disease
- link to the website of the parents' association / foundation / support group
- contact to parents' association / foundation / support group
- contacting a doctor dealing with ALS treatment (including experimental ones)
- I do not remember
- Other:

B.10. What kind of information did you receive during the interview during which the diagnosis was delivered?

- about the mechanism and causes of the disease
- about the treatment options for ALS
- about rehabilitation (physiotherapy, speech therapy, neurological rehabilitation)
- about the options of psychological support
- about the options of social security assistance
- about the domestic ventilation program
- on experimental therapies based on stem cells
- about experimental drug therapies
- on how to deal with the disease in everyday life
- None of the above
- Other:

B.11. In retrospect, do you think that some information that would be helpful was missing?

- Yes (insert below)
- No Go to question 12.

……………………………………………………. ……………………………………………………. …………………

… ……………………………. ……………………………………………………. …………………………………

……… …………. ……………………………………………………. …………………………………………

…. ……… ……………………………………………. ……………………………………………………. ……………

……………. ……………………. ……………………………………………………. ……………………………

…………………… …. ……………………………………………………. ……………………………………………

B.12. When do you think people with ALS should be informed about their disease:

- only after getting a complete diagnosis
- in a situation where the suspicion of disease is well-grounded
- whenever there is a suspicion of the disease

B.13. Please share any other comments and thoughts on the manner in which you received the diagnosis.

……………………………………………………. ……………………………………………………. …………………

… ……………………………. ……………………………………………………. …………………………………

……… …………. ……………………………………………………. …………………………………………

…. ……… ……………………………………………. ……………………………………………………. ……………

……………. ……………………. ……………………………………………………. ……………………………

…………………… …. ……………………………………………………. ……………………………………………
